# Investigation of switching mechanism in HfO_x_-ReRAM under low power and conventional operation modes

**DOI:** 10.1038/srep39510

**Published:** 2016-12-21

**Authors:** Wei Feng, Hisashi Shima, Kenji Ohmori, Hiroyuki Akinaga

**Affiliations:** 1 Nanoelectronics Research Institute (NeRI), National Institute of Advanced Industrial Science and Technology (AIST), Tsukuba, 305-8560, Japan; 2 Faculty of Pure and Applied Sciences, University of Tsukuba, Tsukuba, 305-8573, Japan

## Abstract

Low-power resistive random access memory (LP-ReRAM) devices have attracted increasing attention owing to their advantages of low operation power. In this study, a vertical-type LP-ReRAM consisting of TiN/Ti/HfO_2_/TiN structure was fabricated. The switching mechanism for LP-ReRAM was elucidated as the conductive filament mechanism for conventional mode, and an interface-type switching mechanism for low power mode was proposed. The analysis of low frequency noise shows that power spectral density (PSD) is approximately proportional to 1/*f* for conventional operation mode. Nevertheless, for low power mode, the PSD of low resistance state (LRS) is proportional to 1/*f*, while that of high resistance state (HRS) is clear proportional to 1/*f*^2^. The envelope of multiple Lorentzian spectra of 1/*f*^2^ characteristics due to different traps reveals the characteristics of 1/*f*. For HRS of low power mode, a limited number of traps results in a characteristic of 1/*f*^2^. During the set process, the number of oxygen vacancies increases for LRS. Therefore, the PSD value is proportional to 1/*f*. Owing to the increase in the number of traps when the operation mode changes to conventional mode, the PSD value is proportional to 1/*f*. To the best of our knowledge, this is the first study that reveals the different noise characteristics in the low power operation mode from that in the conventional operation mode.

As the scaling for conventional flash memories approaches technical limitation, alternative device structures such as phase-change memory^1^, resistive random access memory (ReRAM)^2^,^3^, and conductive-bridging random access memory (CBRAM)^4^ are proposed. Among these technologies, ReRAM is a promising fast switching and low power consumption technology^5^,^6^,^7^,^8^. A large amount of research has focused on the origin of the switching mechanism of ReRAM^9^,^10^,^11^,^12^. Although various switching behaviors and different conductive mechanisms are involved in the field, the resistive switching effects can be roughly classified into filament type and interface type according to their conducting behavior in low resistance states. The ionized oxygen vacancies are displaced driven by the electric field, resulting in the formation of a conductive filament (CF) through an insulating layer^13^,^14^,^15^,^16^. Subsequently, different resistance states can be achieved by the formation and disconnection of the CF between two electrodes. This assumption can be verified by examining the low frequency noise^17^,^18^. For both high resistance state (HRS) and low resistance state (LRS) of ReRAM, the power spectral density (PSD) normalized by current in the frequency domain (*f*) is proportional to 1/*f*^2^. As a single-trap Lorentzian function is proportional to 1/*f*^2^, HRS with a limited number of traps results in a slope α ~ 2. For LRS, the number of traps near the conductive path increases. Therefore, the overlap effect of multiple traps near the CF leads to a slope α ~ 1^17^. For CBRAM, the switching mechanism, i.e., filament growth, has been observed using *in situ* STEM (scanning transmission electron microscopy)^19^. For observation of CF, the electron beam absorbed current (EBAC) can be used as a quick and effective method to identify different resistance levels^20^. The direct observation of CF in ReRAM using the EBAC supports the CF mechanism for ReRAM^21^.

In the era of information explosion, significant consumption of energy related to the IT technology has become a serious concern^22^. In order to further decrease the power for operation, memory with operating current less than 1 μA is developed, and is referred as low-power ReRAM (hereinafter LP-ReRAM)^23^,^24^. Govoreanu *et al*. reported a self-rectifying and self-compliant ReRAM cell, which exhibits low operating currents^25^. With the deposition of the HfO_x_ thin film at the sidewall, the HfO_x_-based vertical ReRAM was fabricated showing low currents and high switching speed^26^. Shima *et al*. previously found that the reliable bipolar resistance state (RS) can be demonstrated in the TiN/Ti/HfO_2_/TiN ReRAM structure by introducing post deposition annealing (PDA) process^27^. This result implies that the control of the redox reaction in the ReRAM cell is crucial to obtain the optimum operating performance of this memory. In particular, the redox reaction at the Ti/HfO_2_ interface and the oxygen concentration profile affect the operating performance. Regarding the switching mechanisms of LP-ReRAM, it is reported that the resistance is inversely proportional to the cell area suggesting an area type, rather than filamentary switching mechanism^25^. However, the switching mechanism of LP-ReRAM was not clearly understood.

In this study, we successfully reduced the memory operation power in TiN/Ti/HfO_2_/TiN ReRAM. Conventional resistive switching requires the assistance of Joule heating in addition to the electric field because considerable amount of oxygen ions around the filament need to diffuse long distance to fill the defects. On the other hand, limited number of oxygen ions near the Ti/HfO_2_ interface contributes to the low-power resistive switching. Moreover, in this latter case, the electric field becomes the dominant driving force for the oxygen ion movement. The operating current can be lower than 0.1 μA, and the interface-type RS is plausible as the mechanism for this low power mode. We studied the switching mechanism for low power mode and conventional mode of LP-ReRAM. In order to substantiate that the switching mechanism is the CF switching mechanism or the interface switching mechanism, the direct observation for CF under two operation modes was performed using EBAC. Moreover, we illustrate the transition of switching mechanism from the possible interface-type under low power mode to the conventional filamentary-type. In order to further understand the switching mechanism of LRS and HRS under two operation modes, the measurements of low frequency noise were carried out. The noise characteristics were examined for LRS and HRS under two operation modes of LP-ReRAM.

## Results

### Resistive switching behaviors of LP-ReRAM

**Figure 1:**
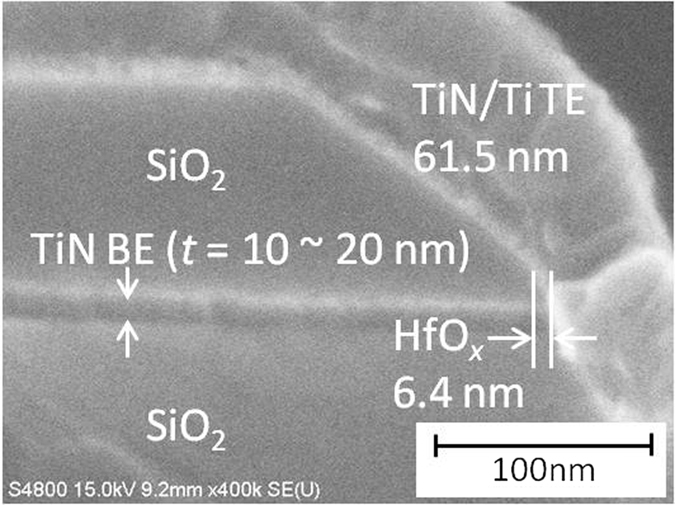
SEM images of cross-section for LP-ReRAM with vertical structure. The thickness *t* is 10–20 nm for TiN, and 6.4 nm for HfO_x_. The deposition rate on the sidewall of the device to the flat part is approximately 70%.

**Figure 2:**
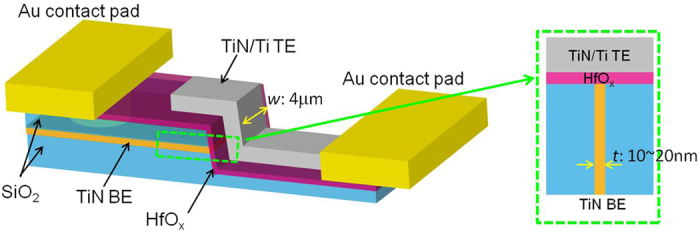
Schematic image of LP-ReRAM with the sidewall of the SiO_2_/TiN/SiO_2_ tri-layer as the bottom electrode (BE).

The cross-sectional SEM image of the LP-ReRAM and the corresponding schematic illustration are shown in Figs 1 and 2, respectively. In this study, we fabricated a vertical-type TiN/Ti/HfO_x_/TiN ReRAM structure. During the fabrication, the thickness of TiN BE is easily controlled using the deposition process. The vertical-type structure is advantageous from the viewpoint of increasing memory density. The vertical-type memory can be integrated, for example, on the sidewalls of deep via holes. The sidewall of the SiO_2_/TiN/SiO_2_ tri-layer was used as the bottom electrode (BE). After sputtering the HfO_2_ blanket, the top electrode (TE) line of TiN/Ti was fabricated by the conventional photolithography. As shown in Fig. 2, the thickness of TiN BE (*t*) and the width of TiN/Ti TE (*w*) define the size of the memory cell size as *t* × *w*. The Au/Ti contact pads were fabricated in order to conduct the electrical measurements. Finally, the PDA process was introduced at 350 °C in the Ar atmosphere (see Methods section).

**Figure 3:**
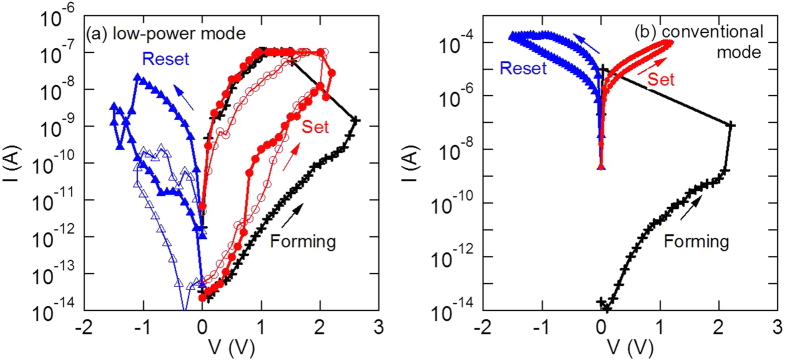
Typical IV curves showing resistive switch observed in LP-ReRAM under two operation modes as (**a**) low power mode with current less than 1 μA, and (**b**) conventional mode with a resistance window of around 10 for both operation modes.

The typical bipolar RS current–voltage (I–V) curves of LP-ReRAM with size 20 nm × 4 μm in two operation modes are shown in Fig. 3(a) and (b). The current level under low power mode is less than 1 μA, and less than 100 μA under conventional mode. The resistance window is around 10 for two operation modes. The low power or conventional operation mode of the device is controlled by the current compliance (CC). Even if the applied voltage value is almost the same, the device that allowed a significant current flow tends to show the conventional switching. One more set of I–V curves of LP-ReRAM with same size is added as hollow symbols in Fig. 3(a), showing a significant variation on the resistance value in LRS as well as the current value required for the reset process. As the variation of the resistance value in LRS is significantly large, and it is affected by the value of compliance current, it is difficult to discuss the device size dependence. Furthermore, note that the oxygen-vacancy density depends on several factors, such as the current compliance, the shape of the device, and the heterogeneity degree of the oxide layer. Therefore, the possibility of nonconstant oxygen-vacancy density with decrease in the device size makes it difficult to discuss the device size dependence.

### CF observation using EBAC

**Figure 4:**
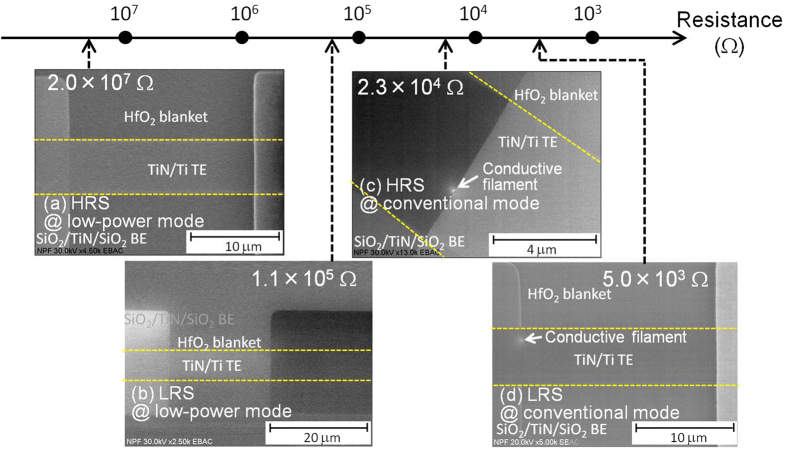
EBAC images of LP-ReRAM at (**a**) HRS and (**b**) LRS of low power mode, (**c**) HRS and (**d**) LRS of conventional mode. A CF was observed at conventional mode, indicated by an arrow.

According to their conducting characteristics in LRS, the resistive switching effects can be roughly classified into two types: the filament type and the interface type. In order to determine the CF switching mechanism and interface switching mechanism for LP-ReRAM, the direct observation of CF using EBAC was performed at HRS and LRS under two operation modes (see Methods section). The gradations of light and dark are shown in the EBAC images. In this grayscale image, the bright area indicates the low resistance portion as shown in Fig. 4. The area of TiN, where the conductive filament is expected to be visible, is shown between the yellow dashed lines. As shown in Fig. 4(a) and (b), the CF was not observed at HRS and LRS under low power operation modes. In contrast, after switching from low power mode to conventional mode, the CF area at HRS and LRS of conventional mode is clearly observed, and is indicated by an arrow in Fig. 4(c) and (d). With direct observation using EBAC, it was confirmed that CF was not formed in LRS under low power mode. Therefore, we proposed a possible interface-type switching mechanism for low power mode. When CF was formed by an adequate number of oxygen vacancies, the LP-ReRAM changed from low power mode to conventional mode. Once the CF was formed, the LP-ReRAM works under conventional mode, and does not return to the low power mode.

## Discussion

**Figure 5:**
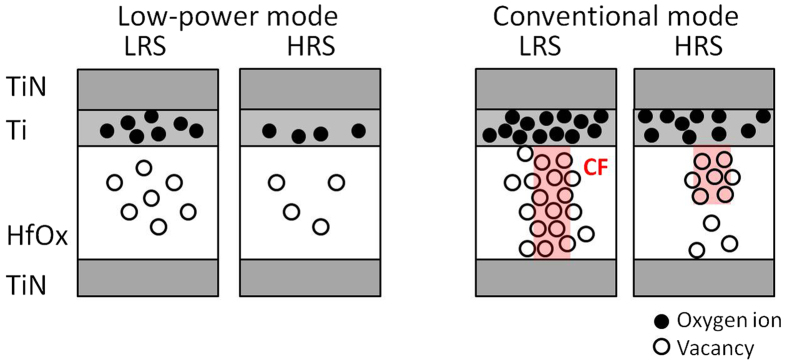
Switching mechanism of LP-ReRAM at LRS and HRS under two operation modes.

A CF-type switching mechanism of LP-ReRAM was observed in the conventional mode. As confirmed by EBAC observation, a metallic filament is formed at LRS, as shown at the “Conventional mode” part in Fig. 5. CF was not observed by EBAC, which is in support of an interface type switching mechanism for low-power mode. The limited number of oxygen ions near the Ti/HfO_2_ interface contributes to the resistive switching under low power mode.

**Figure 6:**
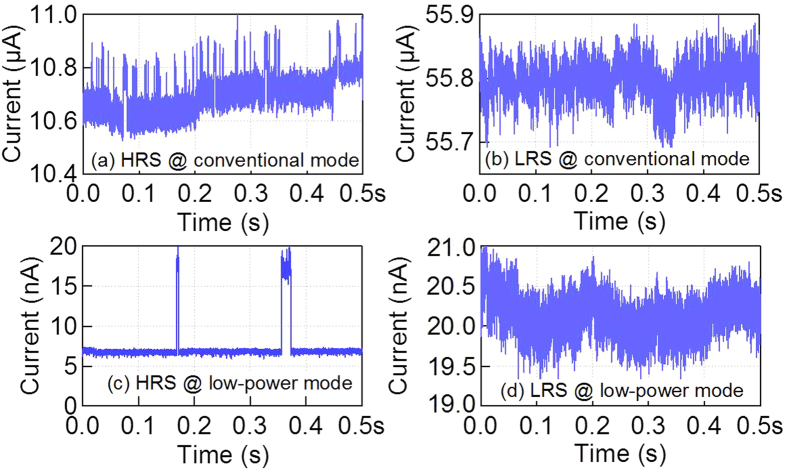
Current fluctuations in the time domain for different resistance states at room temperature. The DC voltage bias for read-out is 0.2 V for conventional mode. For low power mode, the read-out voltage is 0.4 V for adequate current level around 10 nA to measure.

In order to further understand the switching mechanism of LP-ReRAM, the low frequency noise was examined by measuring the current fluctuation using the waveform generator/fast measurement unit (WGFMU) for low frequency noise analysis (see Methods section). The current value at time domain for LRS and HRS under two operation modes was shown in Fig. 6. With read-out voltage as 0.4 V, the current is at nA level under low power mode, and μA level under 0.2 V of conventional mode. The LP-ReRAM exhibits random telegraph noise (RTN) under HRS of both conventional operation and low power modes (Fig. 6(a) and (c)), whereas no clear RTN was observed under LRS (Fig. 6(b) and (d)). For conventional operation mode, the RTN was clearly observed under HRS. The various levels of current as a result of capture/emission process indicated multiple dominant traps. As the current mainly flows through the wider and stable CF under LRS, the capture/emission process was not obvious to detect, and the current fluctuation was smaller than that of HRS. Under low power mode, the clear RTN for HRS was observed at nA level. The two main current levels indicate one dominant trap. Because the number of traps increases during set process, the RTN signals percolate with each other, forming a continuous noise current data as that of LRS under low power mode.

**Figure 7:**
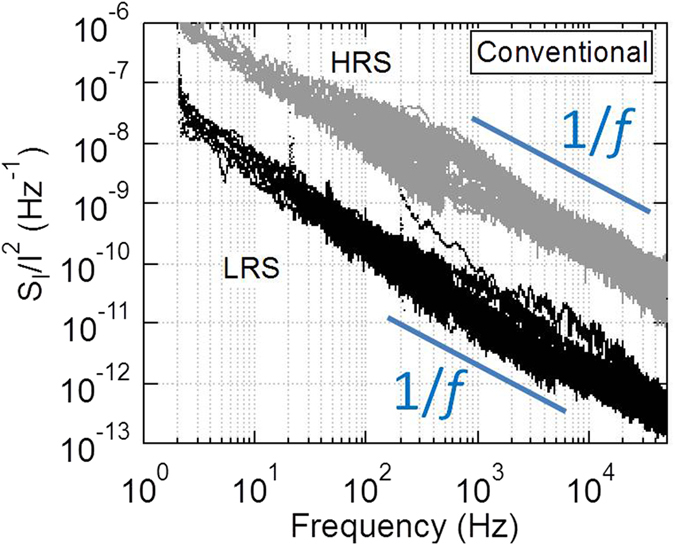
Normalized PSD (S_I_/I^2^) for different resistance states of conventional mode. For both LRS and HRS, the slope α value is close to 1.

The PSD analysis was performed for current fluctuation of LRS and HRS under two operation modes. The normalized PSD value was obtained by dividing the PSD value with the square of the average current. As the CF mechanism is better understood from research^11^,^13^, the switching mechanism under conventional mode is first discussed. The normalized PSD value under conventional mode is shown in Fig. 7. The normalized PSD value of HRS is proportional to 1/*f*^α^ with α value as 1.3 ± 0.2 as a result of multiple dominant traps are shown as an example in Fig. 6(a). Note that there is noise data showing 1/*f*^2^ characteristics for HRS among 10 set measurements. The envelope of multiple Lorentzian spectra due to different traps reveals the characteristics of 1/*f*. The α value of LRS as 1.0 ± 0.2 is smaller than that of HRS, because of the increase in the number of traps during the set process. Similar characteristics have been reported in other studies^28^. The level of normalized noise is about two orders of magnitude larger in HRS than in LRS, which is similar to the results reported in ref. 29.

**Figure 8:**
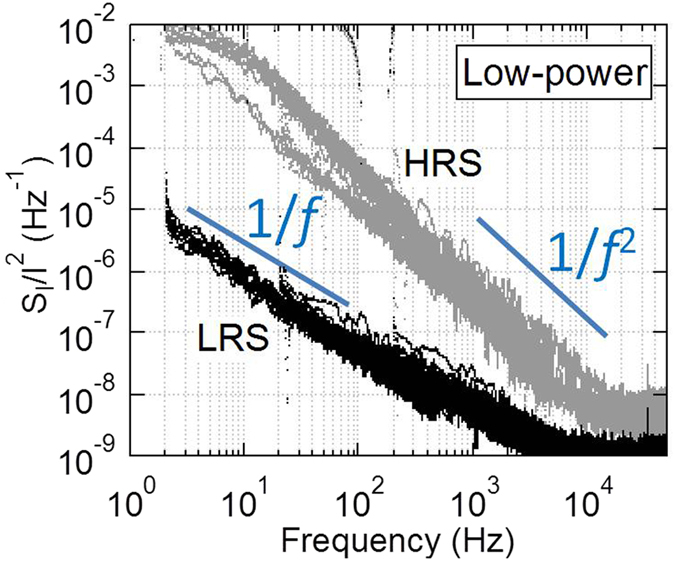
Normalized PSD (S_I_/I^2^) for different resistance states of low power mode. From HRS to LRS, the slope α changes from 2 to 1 owing to the increase in the number of traps during the set process.

For low power operation mode, the normalized PSD values of two resistance states are shown in Fig. 8. The normalized PSD value was larger than that of conventional mode with value up to 10^−3^ Hz^−1^. The level of normalized noise is larger in HRS than that in LRS. The normalized PSD value of HRS shows clear proportional to 1/*f*^2^ due to one dominant trap as shown in an example in Fig. 6(c). We proposed a possible model that LRS showed the characteristics of 1/*f* as the number of oxygen vacancies increases for LRS from HRS during the set process under low power mode. When the oxygen vacancies increase to form a CF for changing the operation mode to conventional mode, the number of traps increases. Different noise behavior was observed for low power mode from that under conventional operation mode.

**Figure 9:**
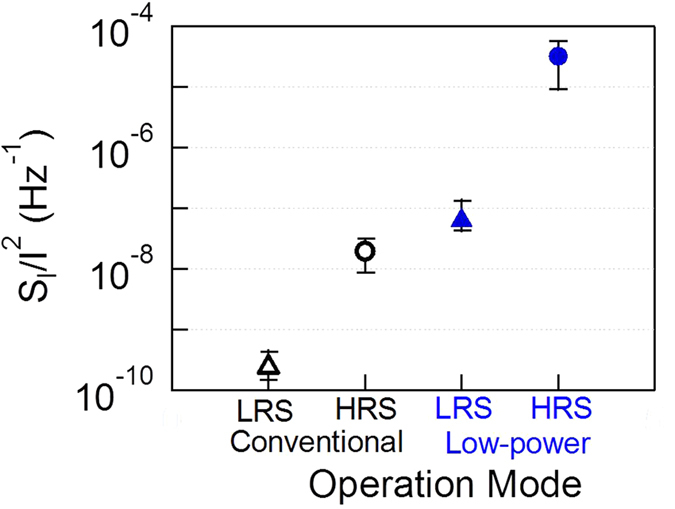
Comparison of normalized PSD (S_I_/I^2^) values at 100 Hz of LRS and HRS between different modes.

The normalized PSD value at 100 Hz of 10 sets of measurement data is shown in Fig. 9. The average value of the normalized PSD value under low power mode is two orders larger than that of conventional mode for each resistance state. For both operation modes, the noise level of HRS is two orders larger than that of LRS.

In conclusion, we investigated the switching mechanism of HfO_x_-based LP-ReRAM. The CF was formed at the LRS of the conventional mode, which was directly observed using EBAC. Moreover, the possible switching mechanism of low power mode was revealed to be an interface type as CF was not observed by EBAC. To the best of our knowledge, this is the first study revealing the different noise characteristics for low power mode from that under conventional mode. The normalized PSD value of HRS under low power mode is proportional to 1/*f*^α^ with α value close to value 2. A possible reason is that a limited number of traps is dominant for low power mode. The number of traps increases during the set process for LRS as well as while changing the operation mode to conventional mode. Then, the characteristics is proportional to 1/*f*, for LRS under low power mode, and the two resistance states of conventional mode, as the envelope of multiple Lorentzian spectra of 1/*f*^2^ caused by different traps. For the first time, we reveal that the noise characteristics of the low power operation mode is different from that of the conventional mode.

## Methods

TiN BE was sputter-deposited using the RF magnetron sputtering method. A mixture of Ar and N_2_ gas was used in the process. Subsequently, the SiO_2_ layer was deposited using the chemical vapor deposition process. To expose the sidewall of TiN BE for the obtained SiO_2_/TiN/SiO_2_ structure, the Ar ion milling and reactive ion etching processes were conducted. This TiN sidewall sandwiched between two SiO_2_ layers was used as the electrode. The HfO_2_ blanket was also sputter-deposited. Then, the TE line of TiN/Ti was fabricated using the conventional photolithography. The deposition of TiN/Ti layers was performed using the RF magnetron sputtering method. The thickness of TiN BE and the width of TiN/Ti TE define the size of a memory cell. The Au/Ti contact pads were fabricated by electron beam evaporation. Finally, the PDA process was introduced at 350 °C in the Ar atmosphere.

The basic resistive switching characteristics of the LP-ReRAM samples were observed using the I–V sweep function of SMU (source/measure unit) in Agilent B1500. The voltage was applied on the TE with BE grounded. The forming voltage is about 3 V with a compliance current of 0.1 μA. The transition for the ReRAM device from HRS to LRS (set) process occurs under positive voltage. On the other hand, the transition from LRS to HRS (reset) process occurs at the opposite direction. Although there is some statistical dispersion during the repetition of RS, the absolute values of set and reset voltages are typically smaller than 2 V. The conventional RS mode was observed when CC was greater than 1 μA.

The EBAC function of Nanoprober N6000 fabricated by Hitachi High-Technologies was utilized to substantiate the CF with a capability to observe a current level of low to 100 pA, which is suitable for observing a current level of around 10 nA for low power mode devices. Following the set process under low power mode, the sample of LRS was put in Nanoprober N6000 to substantiate CF using EBAC. The CF was not observed indicating the possibility that CF was not formed. Then, the sample was taken out for continuing the resistance switch and noise measurements. Then, for the HRS under low power mode, and the LRS and HRS of conventional mode, the sample was put back in Nanoprober N6000 under EBAC observation. For the conventional mode, a clear image of CF was observed, by setting the emission current, I_e_, as 10 μA and normal probe current.

The noise measurements were performed following the set process to LRS and the reset process to HRS for low power mode and conventional mode. For each resistance state, the noise measurements were repeated 10 times. The WGFMU (Agilent B1530A) was employed for monitoring the current fluctuation of LP-ReRAM. The constant dc biases of read-out voltage were applied on the LP-ReRAM samples. In addition, the read-out voltage is set as 0.2 V for conventional mode. In order to have a substantially adequate current level to measure for low power mode, the voltage is set to 0.4 V to have a current level of 10 nA. The sampling rates were set from 10^3^ to 10^5^ s^−1^ and the number of sampling points was 50000. We characterized the noise characteristics of four LP-ReRAM samples, from which one representative sample was shown. The PSD, as the frequency response of measured current values, was calculated. Note that the PSD value seems discontinuous around 500 Hz in Figs 7 and 8, because the calculation of the measured data with sampling rate 10^3^ s^−1^ shows PSD value until 500 Hz at frequency domain.

Device fabrication process was conducted in Nano-processing facility (NPF) in AIST.

## Additional Information

**How to cite this article**: Feng, W. *et al*. Investigation of switching mechanism in HfO_x_-ReRAM under low power and conventional operation modes. *Sci. Rep.* **6**, 39510; doi: 10.1038/srep39510 (2016).

**Publisher's note:** Springer Nature remains neutral with regard to jurisdictional claims in published maps and institutional affiliations.
